# Manufacturing Mesenchymal Stromal Cells for the Treatment of Osteoarthritis in Canine Patients: Challenges and Recommendations

**DOI:** 10.3389/fvets.2022.897150

**Published:** 2022-06-10

**Authors:** Ana Ivanovska, Mengyu Wang, Tarlan Eslami Arshaghi, Georgina Shaw, Joel Alves, Andrew Byrne, Steven Butterworth, Russell Chandler, Laura Cuddy, James Dunne, Shane Guerin, Rob Harry, Aidan McAlindan, Ronan A. Mullins, Frank Barry

**Affiliations:** ^1^Regenerative Medicine Institute (REMEDI), Biosciences, National University of Ireland Galway, Galway, Ireland; ^2^ValleyVets, Cardiff, United Kingdom; ^3^BrayVet, Bray, Ireland; ^4^Orthopaedics, Weighbridge Referral Centre, Swansea, United Kingdom; ^5^Orthopaedic Referral Service, Alphavet Veterinary Centre, Newport, United Kingdom; ^6^Small Animal Surgery, Canine Sports Medicine and Rehabilitation, Veterinary Specialists Ireland, Summerhill, Ireland; ^7^Knocknacarra Veterinary Clinic, Ark Vets Galway, Galway, Ireland; ^8^Small Animal Surgery, Gilabbey Veterinary Hospital, Cork, Ireland; ^9^Northern Ireland Veterinary Specialists, Hillsborough, United Kingdom; ^10^Department of Small Animal Surgery, School of Veterinary Medicine, University College Dublin, Dublin, Ireland

**Keywords:** mesenchymal stromal cells, canine, osteoarthritis, cell therapy, quality control criteria, cell manufacturing, One Health

## Abstract

The recent interest in advanced biologic therapies in veterinary medicine has opened up opportunities for new treatment modalities with considerable clinical potential. Studies with mesenchymal stromal cells (MSCs) from animal species have focused on *in vitro* characterization (mostly following protocols developed for human application), experimental testing in controlled studies and clinical use in veterinary patients. The ability of MSCs to interact with the inflammatory environment through immunomodulatory and paracrine mechanisms makes them a good candidate for treatment of inflammatory musculoskeletal conditions in canine species. Analysis of existing data shows promising results in the treatment of canine hip dysplasia, osteoarthritis and rupture of the cranial cruciate ligament in both sport and companion animals. Despite the absence of clear regulatory frameworks for veterinary advanced therapy medicinal products, there has been an increase in the number of commercial cell-based products that are available for clinical applications, and currently the commercial use of veterinary MSC products has outpaced basic research on characterization of the cell product. In the absence of quality standards for MSCs for use in canine patients, their safety, clinical efficacy and production standards are uncertain, leading to a risk of poor product consistency. To deliver high-quality MSC products for veterinary use in the future, there are critical issues that need to be addressed. By translating standards and strategies applied in human MSC manufacturing to products for veterinary use, in a collaborative effort between stem cell scientists and veterinary researchers and surgeons, we hope to facilitate the development of quality standards. We point out critical issues that need to be addressed, including a much higher level of attention to cell characterization, manufacturing standards and release criteria. We provide a set of recommendations that will contribute to the standardization of cell manufacturing methods and better quality assurance.

## Introduction

Regenerative medicine and stem cell research represent a disruptive technology (1) that brings innovative approaches for the treatment of unmet clinical needs, and an opportunity to study diseases from a novel perspective on a cellular and molecular level. Their disruptive nature is particularly evident in emergence of a new generation of veterinary medicinal products for clinical use. With new opportunities come challenges that affect directly all stakeholders involved in the field, including private and public research institutions, veterinary healthcare professionals, industry and regulatory agencies. The integration of traditional medical and surgical treatments with stem cell therapies is of scientific, clinical and public interest, especially for the treatment of chronic degenerative diseases where currently symptom modifying treatments are the only therapeutic option.

Osteoarthritis (OA) is a major cause of pain, disability and lameness in dogs and current treatment protocols mainly include: non-steroidal anti-inflammatory drugs (NSAIDs), anti-nerve growth factor monoclonal antibodies (2), physical activity moderation or restriction, weight control and nutraceuticals, which aim to alleviate symptoms and disease progress, with surgical treatments being applied in severe cases (3). The significant health and welfare burden of OA on veterinary patients is strongly influenced by the epidemiology of the disease globally. In the United Kingdom, there is an estimated annual prevalence of 2.5-6.6% of dogs of different age and breeds, based on primary veterinary care data (4, 5). The reported prevalence in North America is age-specific, with 20% of patients being over 1 year of age, and 80% of them being over 8 years old (6). The use of cell therapies for the management of OA has shown promising pre-clinical and clinical results in canine patients (7–10). This has given rise to a range of canine MSC products for the treatment of joint disease available on the market, however few are underpinned by robust assessment of safety and efficacy in well-designed clinical trials. Furthermore, the lack of stringent regulatory guidelines for veterinary cell-based products has allowed clinics and companies to operate in a so-called “commercial environment zone” relying on preliminary pre-clinical and clinical data and poorly defined, unstandardised manufacturing protocols. These issues give rise to a number of important concerns. Firstly, the use of unproven cell therapies lacking clinical validation represents a potential risk to the health of veterinary patients. Secondly, the absence of clear and rigorous quality standards for veterinary cell products signifies a possible vulnerability that could impact both manufacturers and practitioners. Thirdly, an unregulated market lacking transparency permits unproven and potentially unsafe therapies to gain traction. Finally, the absence of appropriate pharmacovigilance systems guided by international consensus and oversight, means that adverse events will potentially be unmonitored and unreported.

In this review we discuss the critical processing elements required for the manufacture of canine MSCs to ensure high-quality, well characterized and standardized products. Here, we will target canine OA as a model disease for the cell manufacturing process, being a high prevalence chronic condition with a major impact on animal welfare. We hope to provide the necessary insights and highlight the major aspects of cell production and characterization that need to be considered and improved upon to manufacture high-quality products for canine patients.

## Mesenchymal Stromal Cells

**Figure 1:**
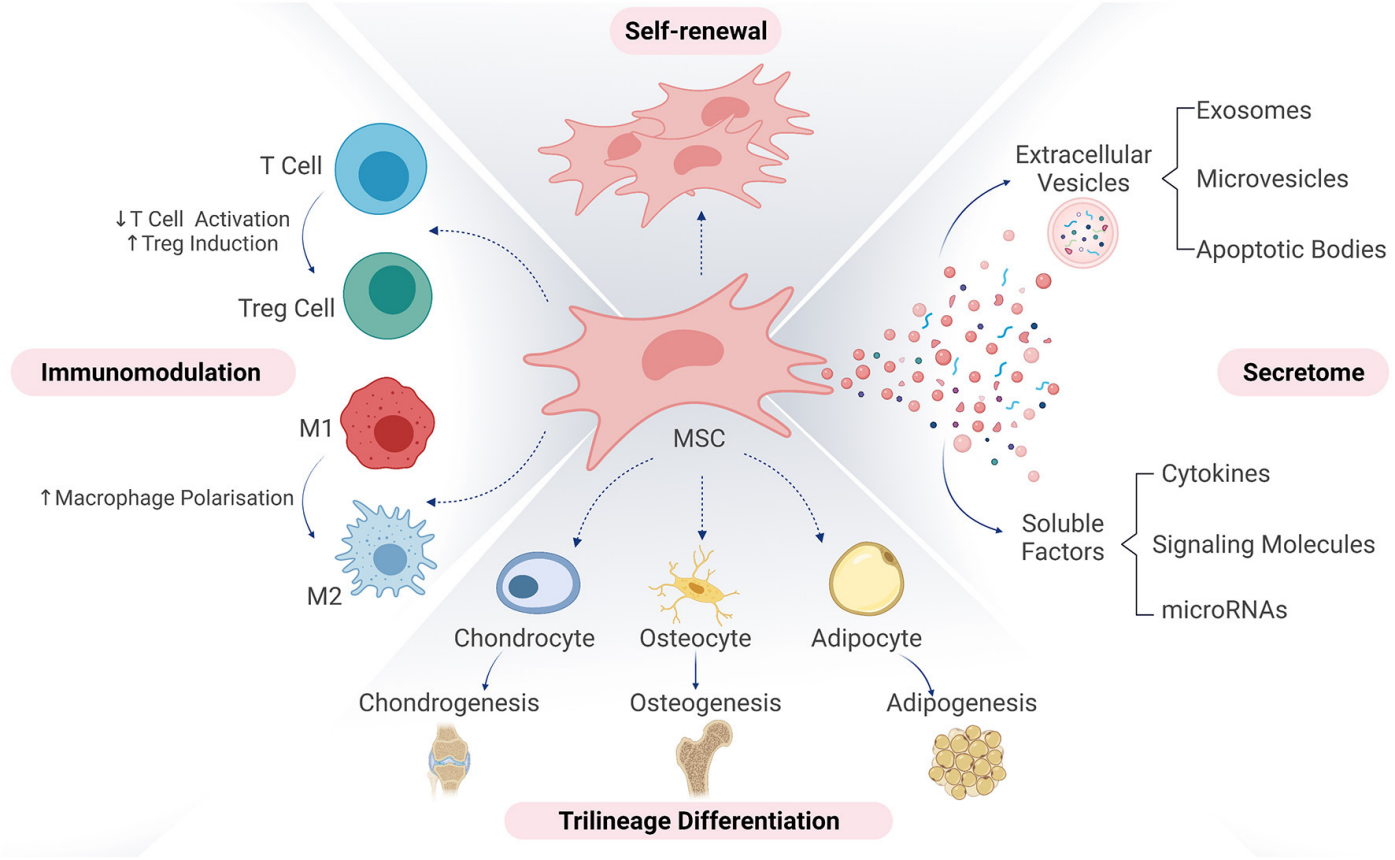
Therapeutic mechanisms of action of mesenchymal stromal cells. MSCs are defined by their ability for self-renewal, proliferation and undergo tri-lineage differentiation into adipogenic, chondrogenic and osteogenic lineages. They contribute to tissue repair *via* multiple proposed mechanisms of actions. These include a direct contribution to tissue repair *via* engraftment and cell differentiation but also immunomodulation of host immune system by counteracting the pro-inflammatory cascades and establishing an anti-inflammatory micro-environment for tissue healing and secretion of extracellular vesicles and soluble factors responsible for intracellular communication with target cells. Treg, regulatory T cell; M1, macrophage type 1; M2, macrophage type 2. Figure created with BioRender.com.

MSCs have been extensively studied *in vitro* and *in vivo* due to their unique trophic, paracrine and immunomodulatory properties (Figure 1). The initial rationale for therapeutic use of MSCs was based on their multipotent nature, (11, 12), which includes their ability to differentiate into multiple cell types. However, studies showing limited cell survival and engraftment upon transplantation (13, 14), accompanied by tissue regeneration suggest a paracrine mechanism of action mediated by secreted factors. MSCs express a set of receptors on their surface facilitating active communication with the diseased environment (15, 16), influencing the immune cell response toward an anti-inflammatory phenotype and secreting immunomodulatory factors to dampen the inflammatory microenvironment (17–20). Additionally, MSCs secrete bioactive molecules with pro-angiogenic, anti-fibrotic, chemotactic and trophic properties, thus promoting tissue regeneration. These secreted factors are divided into two categories, enveloped secretion factors, also termed extracellular vesicles (EVs) and soluble factors. The short survival of MSCs post-injection is believed to be influenced by the harsh environment *in vivo*, which induces cell apoptosis (21, 22). However, this does not hinder their therapeutic properties, as the apoptotic bodies can still exert immunomodulatory properties (23).

Based on this complex network of therapeutic mechanisms, MSCs have the potential to be disease-modifying rather than symptom-modifying compared to conventional medical treatments (24).

**Figure 2:**
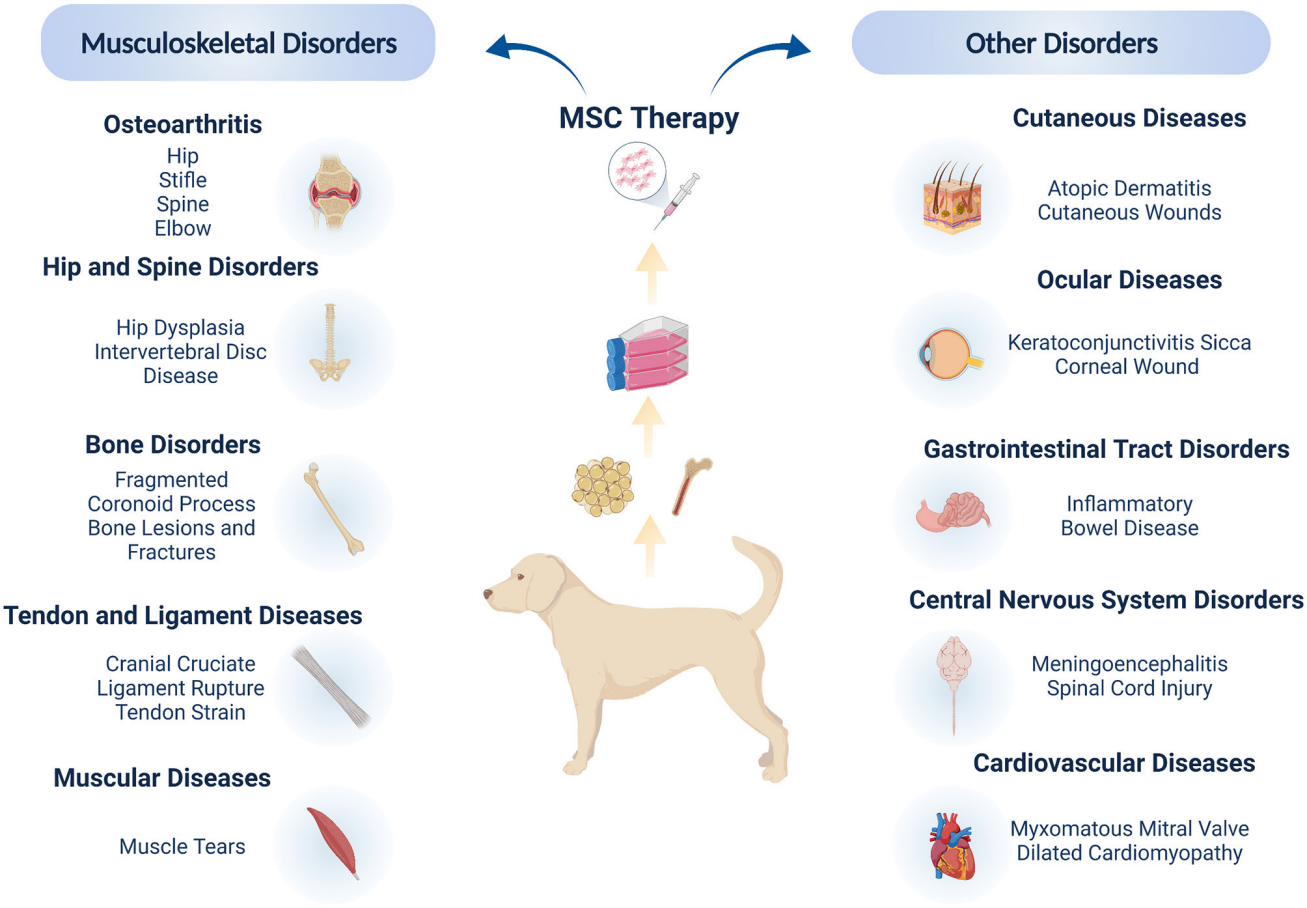
Mesenchymal stromal cells in canine clinical studies. The majority of clinical applications address orthopedic and musculo-skeletal disorders, due to the high incidence of these conditions in dogs coupled with the ease of local administration of MSCs. Clinical trials have also highlighted the local and systemic applications of MSCs for other soft tissue conditions, showing the wide potential of MSC therapies in canine patients. Figure created with BioRender.com.

The clinical application of MSC-based therapy in veterinary medicine comprises of multiple target diseases, with the vast majority being focused on musculoskeletal conditions such as osteoarthritis (8, 25–34), cruciate ligament disease (9, 35–38), hip dysplasia (39), bone fractures and lesions (40, 41), muscle tears (42, 43) and tendinopathies (44, 45). Canine clinical studies have also shown promising results for the treatment of diseases of the gastro-intestinal tract (46–48), central nervous system (49–53), cardiovascular system (54, 55), cutaneous (56–59) and ocular conditions (60–63), resulting in encouraging safety and efficacy data (Figure 2).

## Cell Manufacturing For Veterinary Clinical Use

### Autologous and Allogeneic Cell Products

**Figure 3:**
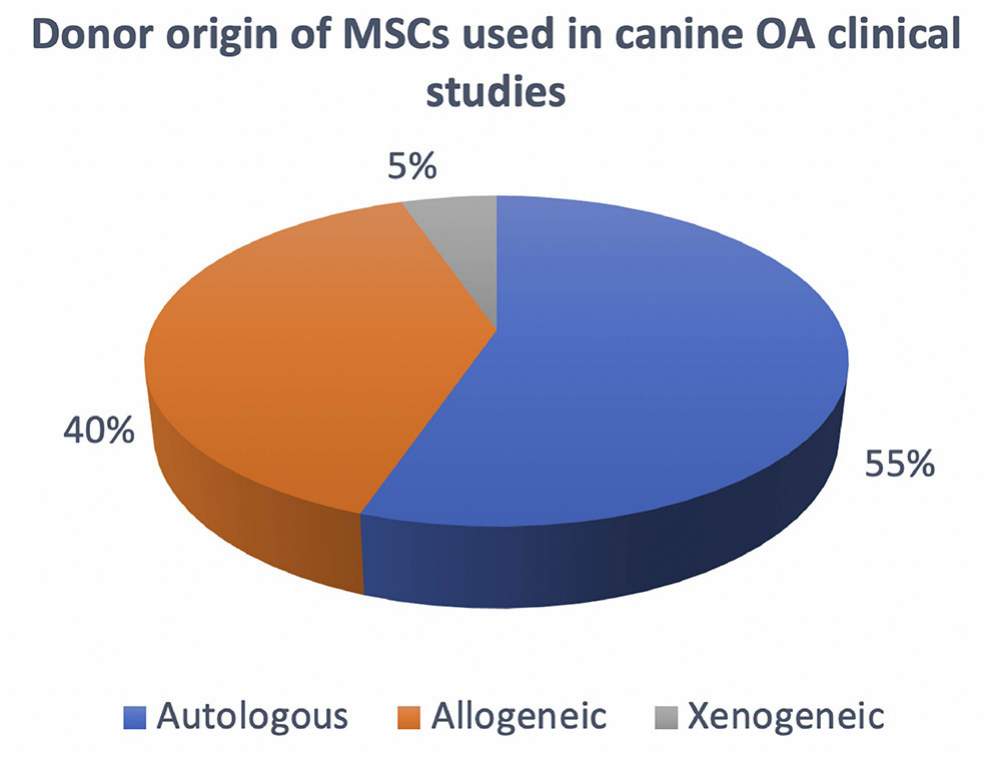
Overview of the current use of autologous, allogeneic and xenogeneic cells in canine OA clinical studies.

Based on the donor origin of MSCs, these can be classified as autologous if the donor and patient are the same individual, allogeneic if the donor and patient both belong to the same species but are genetically and immunologically different, while if they both belong to different species they are termed xenogeneic. The cell manufacturing process differs whether the product being manufactured for administration is for allogenic or autologous use. The differences in terms of advantages and disadvantages are also important on a clinical and therapeutic level, however this will not be considered in this review since it has already been published and discussed previously (64, 65). In terms of cell manufacturing for osteoarthritis, it is important to take in consideration both autologous and allogeneic products. Based on the published literature on canine OA clinical studies there seems to be an almost equal use of both products (Figure 3). Autologous products seem to be used in a slightly higher percentage, however taking in consideration the timelime of the published literature, the most recent studies have been focusing on allogeneic products as a preferred future therapeutic direction (Table 1). Here throughout the manufacturing process we will take into consideration both autologous and allogeneic products, highlighting specific aspects that need to be considered for each respectively.

**Table 1:** The most common tissue and donor sources for MSCs used in canine OA clinical studies.

**Tissue**	**MSCs based on donor origin**	**Year**	**References**
AT	Auto-MSCs	2007	(7)
AT	Auto-MSCs	2008	(25)
AT	Auto-MSCs	2012	(27)
AT	Auto-MSCs	2012	(66)
AT	Auto-MSCs	2013	(67)
AT	Auto-MSCs	2014	(68)
AT	Auto-MSCs	2014	(35)
AT	Auto-MSCs	2014	(39)
AT	Auto-MSCs	2014	(69)
AT	Xeno-MSCs	2014	(70)
AT	Auto-MSCs	2014	(71)
AT and BM	Auto-MSCs	2016	(72)
AT	Allo-MSCs	2016	(8)
AT	Auto+allo-MSCs	2016	(73)
AT	Auto-MSCs	2016	(74)
BM	Auto-MSCs	2016	(37)
AT	Auto-MSCs	2016	(75)
AT	Auto-MSCs	2016	(28)
AT	Allo-MSCs	2017	(76)
P	Allo-MSCs	2017	(9)
AT	Auto-MSCs	2018	(77)
AT	Allo-MSCs	2018	(29)
AT	Auto-MSCs	2018	(78)
AT	Auto-MSCs	2018	(30)
P	Allo-MSCs	2019	(31)
PB	Xeno-MSCs	2019	(79)
AT	Allo-MSCs	2019	(32)
UC	Allo-MSCs	2019	(80)
AT	Allo-MSCs	2019	(33)
NK	Allo-MSCs	2019	(81)
AT	Allo-MSCs	2020	(82)
AT	Allo-MSCs	2020	(83)
AT	Allo-MSCs	2020	(34)
AT	Auto-MSCs	2020	(26)
AT	Allo-MSCs	2020	(84)
AT	Allo-MSCs	2021	(85)
AT	Auto-MSCs	2021	(86)

*AT, adipose tissue; BM, bone marrow; P, placenta; PB, peripheral blood; UC, umbilical cord; NK, not known; Auto, autologous; Allo, allogeneic; Xeno, xenogeneic; MSCs, mesenchymal stromal cells*.

### Starting Raw Materials of Animal Origin

**Figure 4:**
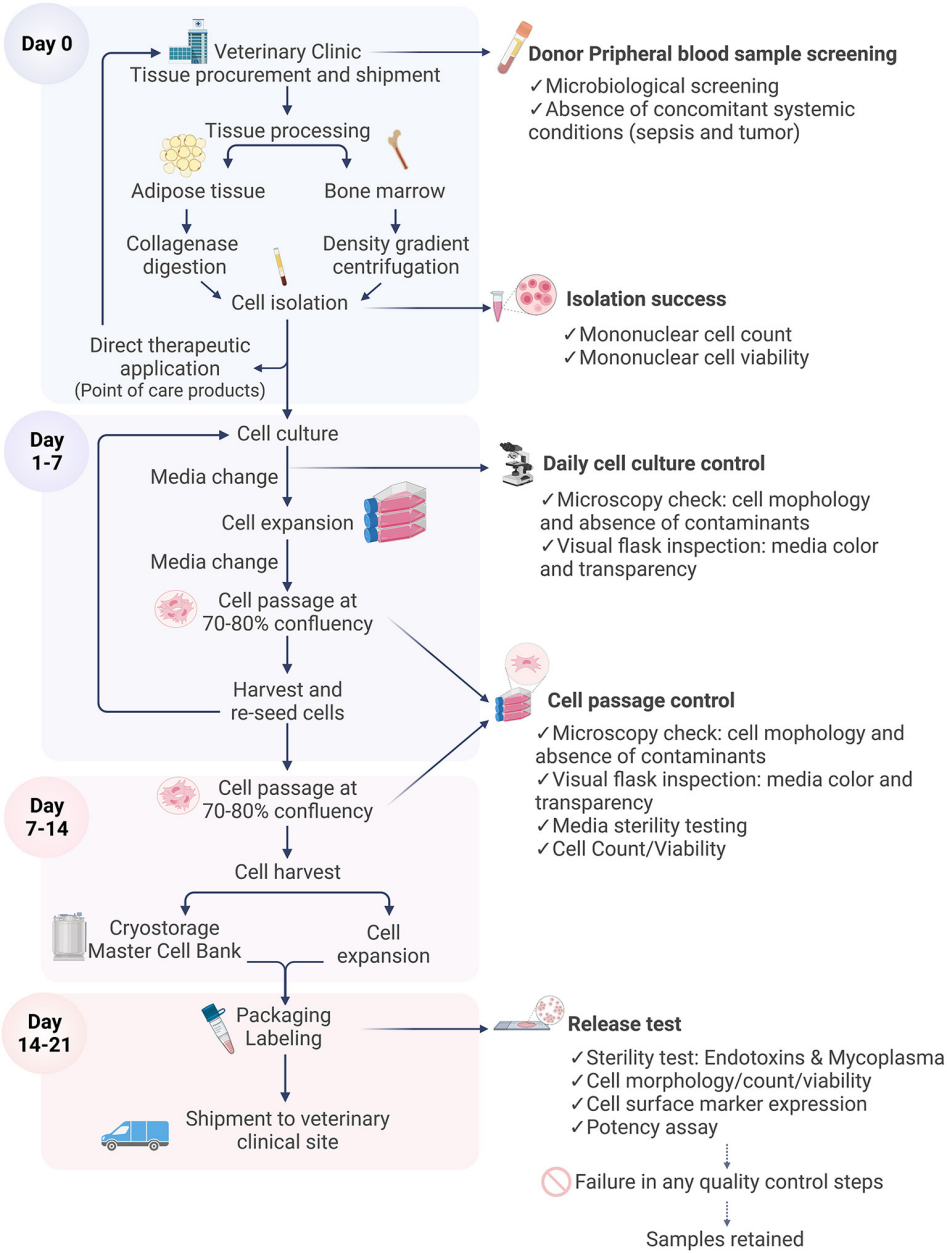
Flow diagram illustrating cell manufacturing stages. The cell manufacturing process starts with tissue sampling at the veterinary clinic. Each stage is critical and is subject to quality control and sterility testing prior to shipment of the final characterized cell product for therapeutic application. In the case of failure of any quality control step, the batch is discarded and in the final stages the sample is retained. Figure created with BioRender.com.

The manufacture of MSCs for clinical application is a complex multi-step process (Figure 4), bearing in mind that the therapeutic product is composed of living cells that cannot be terminally sterilized and filtered. Therefore, the aseptic nature, quality and safety of the tissue source and raw materials used for isolation and production of MSCs have a great impact on the manufacturing process. All donors and tissues should be subject to screening procedures for infective agents to avoid their introduction into the manufacturing process. The Committee for Medicinal Products for Veterinary Use (CMPV) of the European Medicine Agency (EMA) (87), published the following guidelines on screening tissue donors for allogeneic cell products for the following bacterial strains (*Brucella canis, Leptospira* spp., *Borrelia* spp, *Ehrlichia* spp., *Bartonella vinsonii, Anaplasma* spp, *Neorickettsia* spp and *Rickettsia* spp), viral (Canid herpesvirus, Canine adenovirus, Canine coronavirus, Canine distemper virus, Canine oral papilloma virus, Canine parainfluenza 2 virus, Canine parvovirus, Rabies virus and Swine herpesvirus 1) and parasitic pathogens (*Babesia* spp, *Leishmania* spp, *Trypanosoma cruzi, Neospora caninum* and *Dirofilaria immitis*). The protocols for molecular or serological screening for the presence of these pathogens should be efficient and subjected to scrutiny on a case-by-case approach based on detailed medical history and clinical examination relevant to the donors' geographic location. Whilst donor screening is essential, there is still a risk of transmission of infective pathogens. For tissue procurement and manufacturing purposes however, tissues are usually harvested in a controlled operating theater environment which minimizes air-borne contaminants. Well trained and experienced veterinary surgeons should be involved in the collection of the source tissues, using defined and standardized protocols for labeling, storage (in sealed containers) and shipment to the cell production facility.

### Tissue Sources

In veterinary medicine the most commonly used tissues sources of canine MSCs are adipose tissue and bone marrow (Table 1). Bone marrow (BM) was the first described source of canine MSCs where the cells were defined based on their *in vitro* and *in vivo* properties, paving the way to future characterization studies (11). However, from a clinical point of view, bone marrow harvesting is a technically challenging process because of the training and level of expertise needed to access the marrow through the periosteum and cortical bone whilst applying vacuum pressure to physically aspirate bone attached MSCs (88). Bone marrow aspirates (BMA) are usually collected from the iliac crest, proximal humerus and proximal tibia using heparinised syringes, yielding collection volumes of up to 20 ml of marrow (89–93), depending on the size of the patient. From a technical perspective, it has been demonstrated that smaller volumes are more efficient in obtaining a higher concentrations of progenitor cells, because excessive volumes can result in blood dilution of the sample with a marginal increase of bone marrow progenitor cells (88). Bone marrow collection carries a risk of several complications such as hemorrhage, sepsis, pain and infection (94). Cells are isolated by a density gradient centrifugation of the collected BM (95), producing a concentrated heterogeneous cell population of bone marrow aspirate concentrate (BMAC). Bone marrow is predominantly rich in hematopoietic progenitor and stem cells, while mesenchymal progenitors are present in low numbers ranging between 0.001 and 0.02% (96). The administration of BMAC therefore provides a heterogenous population of progenitor cells and includes endothelial progenitors that are able to secrete pro-angiogenic factors and improve the vascularisation of the damaged tissue (97).

Adipose tissue (AT) has become the most common source of canine MSCs due to its high cell yield (98), simplicity in acquiring, and sampling accessibility from surgical wounds and during routine procedures such as ovariohysterectomy (99). In terms of tissue origin AT can be harvested from thoracic, abdominal and inguinal subcutaneous tissue, which could yield a variable volume of tissue based on the body condition score (BCS) of the animal, or from perivisceral adipose tissue like the falciform ligament, omentum and peri-ovarian tissue with ranging yield between 1–30 g (99–103). In terms of sampling locations, subcutaneous AT collected from the inguinal and thoracic wall had a higher yield of cells compared to the falciform ligament. Yield was also significantly higher in spayed females and castrated male dogs, in comparison to intact dogs, showing the importance of age, gender, medical history and sampling location for the MSC yield (100). MSCs are isolated by enzymatic digestion of AT using collagenase, dispase or trypsin for 30-60 minutes of agitation at 37°C degrees (104). The obtained pellet contains the stromal vascular fraction (SVF), a concentrated heterogeneous cell population composed of endothelial cells, lymphocytes, fibroblasts, monocytes/macrophages and erythrocytes (105). The degree of vascularisation of the AT processed could influence the composition. From one perspective a higher percentage of blood vessels would produce more heterogenous range of cell types, however MSCs are known to be located in the perivascular niche and this location could produce a higher overall MSC yield. However, in order to isolate MSC properly it is important to avoid excessive blood contamination of the sample and discard major vessels.

The heterogeneous cell preparations, SVF and BMAC obtained follow tissue processing, can be readily injected into patients as a point-of-care product characterized by minimal *in vitro* manipulation where the injured tissue can benefit from the MSCs, immune and endothelial cells for repair and regeneration (106). In the autologous setting, this approach is commonly used owing to its relative simplicity and the opportunity to perform it completely within the operating theater, thus reducing any risk of contamination and the need for time-consuming cell expansion to obtain sufficient cells. For further therapeutic use aiming to generate a homogeneous cell population the cells are seeded and further expanded. In veterinary medicine, where practical and cost-effective solutions may influence therapeutic choices, cell preparations such as SVF and BMAC offer certain hands-on advantages. These allow for patients to receive cell therapies promptly, whilst reducing time-, cost-, and regulatory-related elements of the cell manufacturing process for culture-expanded MSCs. In certain circumstances point-of-care products can be produced using small medical devices (30), or in house if the veterinary hospital is equipped with needed instruments and facilities. These cell preparations can be administered readily in the operating theater avoiding multiple procedures for the patients. For example, administration of SVF during an orthopedic procedure with the aim to enhance fracture healing and implant integration would benefit greatly from a mixed population of cells like SVF (75, 107, 108). In case of repeated administrations, in older patients or patients affected by systemic conditions allogeneic off-the-shelf products remain the optimal choice. The final choice of which type of cell preparation needs to be administered can be decided on a case to case basis since both offer advantages that could be a better fit in different therapeutic approaches.

Cells from differing tissue sources present different risks of post-transplant morbidity and disease transmission. For example, compared with bone marrow adult derived MSCs, umbilical cord derived MSCs have a lower rate of the risk of viral transmission through transplantation. This could be due to stress-induced activation of latent viruses in adults and a potentially lower rate of infection present at birth in neonatal tissues (87, 109). Similarly, adipose tissue could potentially be exposed to pathogens given its anatomical location in close proximity to the body's physical barriers and lymphoid tissues.

This aspect should be taken in consideration in the choice of autologous or allogeneic products. In case of autologous product the patients' health status is well known and monitored, while donor tissues for allogeneic purposes need to be thoroughly screened as discussed previously.

### Donor Age

Since MSCs for clinical use in dogs are most commonly isolated from adult tissues (Table 1), the age of the donor in terms of metabolic and physiological traits will likely influence the biological properties of MSCs. Specifically for canine MSCs the influence of donor age has been demonstrated to affect negatively certain *in vitro* parameters such as cell yield, colony unit forming abilities, as well as cell differentiation into osteogenic lineage and effect on immune cell modulation (110–112).

In relation to tissue origin, younger dogs have a significantly higher yield of viable cells upon collagenase digestion of fat tissue (100). A similar consideration can be made for bone marrow since its composition changes with aging, when red marrow is replaced by yellow marrow composed predominantly of adipocytes resulting in a lower number of hematopoietic and mesenchymal progenitor cells. However these physiological changes need to be further explored in the canine species to understand better the age-related impact on harvesting bone marrow for cell therapies (113).

In terms of cell characterization, the donor-age effect on CD marker expression has been reported mostly in human MSCs, while few studies have focused on this aspect in canine MSCs. Regardless of donor-age, human AT-MSCs expressed CD44, CD73, CD90 and CD105 and lacked CD3, CD14, CD19, CD34 and CD45 (114–116), while Stolzing and colleagues report age-induced expression of CD44, CD90, CD105 and Stro-1 (117). In canine MSCs, the gene expression of CD90, CD34 and CD45 was similar in young and old donors (118), while Lee et al. (111), reported a significantly higher expression of CD73 and CD80 in younger donors. The intrinsic heterogeneity seems to be induced also by the *in vitro* culture conditions as CD90 and CD44 expression was reduced by P5 and P6 in both young and old donors, indicating that cellular senescence can be induced regardless donor-age (119).

The differences between young and old donors can be attributed to a general age-related metabolic and functional tissue decline, that could affect as well the features of progenitor and stromal cells. It could be suggested that these remaining resident cells would be activated to contribute to restore the homeostasis altered by the aging process and express a different set of surface molecules, compared to young donors where most likely the same cells would be in a quiescent state characterized by a different phenotype.

The donor age also reflects the potential outcomes of autologous and allogeneic therapies. Young donors (5–12 months) would be the most likely chosen for development of allogeneic cell products due to a potential to isolate higher yields of MSCs. Regarding autologous cell products, especially for treatment of OA where older dogs are mostly affected (5) with often impaired healing abilities or other concomitant co-morbidities, it is questionable to what extent will the patient's own cells have a therapeutic effect. In this case to overcome the low yield of MSCs, larger amounts of tissues could be harvested and the resulting cells cultured for longer periods to ensure that adequate cell numbers for treatment are achieved. However, the expansion process needs to be fine-tuned for the optimal delivery of healthy, well-characterized and standardized cells for the intended clinical use.

In terms of up-stream variables, for allogeneic use it is recommended to manufacture MSCs deriving from young donors. For early signs of senescence, it is important to closely monitor the cell morphology as flat and hypertrophic cells are associated with senescence, and to perform colony forming units (CFU-F) assays to analyse the proliferative and clonogenic abilities of MSCs. In addition to these first indicators of potential cell senescence, in process controls including assessing gene and protein expression of senescence-associated markers can be introduced on a batch of cells to monitor their cell cycle dynamics. This can be performed by testing for markers known to be down-regulated in prolonged culture times such as Stro-1 and CD106 (120, 121), in addition to the widely used senescence-associated beta-galactosidase (SA-β-gal) marker (122). Most of the strategies defined here are commonly used for human MSCs and further studies are needed to analyse how these would impact the behavior of canine MSCs.

### Cell Culture

#### Critical Components

In a typical cell expansion process, additives of animal origin are commonly used. These include fetal bovine serum (FBS) and platelet lysates (PLs), which act as a source of nutrients, minerals, essential amino acids and growth factors. Traditionally, FBS is the most common supplement used in cell culture, as it contains high concentrations of embryonic growth promoting factors of fetal blood origin. However, the use of FBS has significant drawbacks. These include the fact that the quality of FBS is variable, with high batch-to-batch differences (123), there is a risk associated with transmissible spongiform encephalopathies (TSE) (124) and the risk of immune reactions in patients treated with cell products (125). Additionally there are ethical concerns related to FBS procurement (126). Stringent control of the geographic origin and quality grade screening by the manufacturers is required, to avoid any impact on the integrity of the product and the clinical outcome (127, 128). FBS is widely used in the manufacture of veterinary cell products that have received market authorization from EMA, due to the fact that equine and canine patients are considered to be species with low susceptibility for TSE (129–131). However, from a regulatory stance, it is recommended that cell manufacturers should, when possible, use non-ruminant materials to minimize risk of transmission of extraneous agents (132). For this reason, PL, in the form of pooled concentrates, has been assessed as an alternative for the expansion of human, canine and equine MSCs (133–136). PL is produced following the mechanical disruption of platelets, during which the content of the alpha granules are released and concentrated to provide a supplement rich in growth factors to facilitate cell expansion (137, 138). Thus PLs contain high levels of many growth factors such as platelet-derived growth factor (PDGF), vascular endothelial growth factor (VEGF), basic fibroblast growth factor (bFGF), insulin-like growth factor (IGF), epidermal growth factor (EGF), and transforming growth-factor beta (TGF-ß) family. The practical advantage of using PL has been demonstrated to be able to promote and sustain growth of human MSCs and may possibly be translated to veterinary species (139). Several studies have shown that PL is a preferred supplement compared to FBS in human MSC expansion (140, 141), with a recent report citing the replacement of FBS with human PL in 77% of the surveyed MSC manufacturing centers in Europe (142). In the case of canine MSCs, contrasting results have been reported and at least one study indicated PL was unable to support growth and proliferation of MSCs, thereby questioning its role in cell isolation and long-term expansion (20), whilst others have shown the ability of PL to be used as a substitute for FBS for expansion of canine MSCs (143). This may be addressed by a greater level of standardization of PL composition and pooling. The difficulties in achieving the needed standardization in canine MSC manufacturing (128) are mostly due to a lack of species-specific reagents and significant canine platelet lysate banks which are needed to generate critical data on quality control.

#### Cell Media Formulations

Cell culture media are designed to provide nutrients, growth factors, minerals and other components that are essential for the growth and proliferation of cells *in vitro*. The advancement of cell culture technology has influenced the composition of cell media, starting from the traditional serum-containing media, that was subsequently substituted with serum-free, protein-free and recently with more sophisticated chemically defined media that would allow a more controlled and standardized environment for cell growth (144). The major effect that different media have on the properties of expanded canine MSCs is listed in Tables 2–4 respectively for Alpha Modified Eagle's Medium (αMEM), Dulbecco's Modified Eagle Medium (DMEM) and serum free media, where a summary of published literature was assessed by identifying the media composition, immunophenotypic profile and tri-lineage differentiation potential.

**Table 2:** Characterization of canine mesenchymal stromal cells cultured in αMEM.

**Culture media**	**Tissue source**	**Seeding density**	**Surface markers**	**Tri-lineage differentiation potential**	**References**
α-MEM+ 10% FBS + 1%pen/strep + 0.05% Fungizone + 0.1 mM ascorbic acid-2-phosphate + 1.00E-0.9M dexamethasone + 1 ng/mL bFGF	BM LV	6x10^3^cells/cm^2^	CD105(+) CD90(+) CD29(+) CD166(+) CD45(−)	AD-CH-OST	(166)
αMEM + 10%FBS + 80 μg/mL gentamicin	AT-OM AT-SUBC	5x10^3^cells/cm^2^	CD90(+) CD44(+) CD45(−) CD11b(−)	AD-CH-OST	(101)
αMEM + 10%FBS + 100 U/ml pen + 100 μg/ml strep	SY BM AT	1x10^2^cells/cm^2^	CD90(+) CD44(+) CD9(+) CD105(+;−) CD45(−) CD34(−) STRO-1(−)	AD-CH-OST	(167)
αMEM + 20%FBS + 100 U/ml pen + 100 mg/ml strep sulfate + 2 mM glutamine	B-MAN B-FEM	NK	NK	AD-CH-OST	(168)
αMEM + 15%FBS+1% pen/strep	AM	NK	CD90(+) CD105(+) CD45(−) CD34(−)	AD-CH-OST	(169)

*αMEM, Alpha Modified Eagle's Medium; FBS, fetal bovine serum; bFGF, basic fibroblast growth factor; Pen, penicillin; Strep, streptomycin; CD, cluster of differentiation; (+), positive expression; (−), negative expression; (+;-), variable expression due to tissue origin; AT, adipose tissue; LV, liver; AT-OM, adipose tissue omentum; AT-SUBC, subcutaneous adipose tissue; SY, synovium; B-MAN, mandible; B-FEM, femur; AM, amnion; AD, adipogenic differentiation; CH, chondrogenic differentiation; OST, osteogenic differentiation; NK, not known*.

**Table 3:** Characterization of canine mesenchymal stromal cells cultured in DMEM.

**Culture media**	**Tissue source**	**Seeding density**	**Surface markers expression**	**Tri-lineage differentiation potential**	**References**
DMEM + 10%FBS	AT BM UCB WJ	1 × 10^5^ cells/cm^2^	CD44(+) CD73(+) CD90(+) CD105(+) CD14(–) CD34(–) CD45(–)	OST	(170)
DMEM + 10% FBS + 1% pen/strep	AT	5 × 10^3^ cells/cm^2^	CD90(+) CD44(+) CD54(+) CD45(–) CD34(–) MHC–II (–)	/	(151)
DMEM-LG + 10%FBS	AM	NK	CD90(+) CD105(+) CD34(–) CD45(–) CD3(–) CD11c(–) CD28(–) CD38(–) CD62L(–) CD41a(–)	AD-CH-OST	(171)
DMEM-LG + 10% FBS + 1% L-glut + 10 ng/ml FGF-β	BM	NK	CD44(+) CD90(+) CD105(+) Vimentin (+)	AD-CH-OST	(172)
DMEM + 10% FBS + 1% pen/strep + 1% L-glut	AT BM	NK	AT: CD90(+) CD44(+) CD29(+) CD8(+/–) CD4(+/–) CD73(+/–) CD 14(–/+) CD34(–/+) CD45(–) MHC–I(+), MHC–II(–) BM: CD90(+) CD44(+) CD29(+) CD8(+/–) CD4(–/+) CD73(+/–) CD 14(–/+) CD34(–) CD45(–) MHC–I(+) MHC–II(–)	AD-CH-OST	(20)
DMEM + 10% FBS + 100 U/ml pen + 100 μg/ml strep + 2.5 mM L-glut + 1.25 μg/ml Fungizone	AD	1 × 10^4^ cells/cm^2^	CD90(+) CD29(+) STRO−1 (+) CD45(–) CD34(–) MHC–II(–)	AD-CH-OST	(60)
DMEM-LG	UCB	NK	CD29(+) CD33(+) CD44(+) CD105(+) CD184(+) Oct−4 (+) CD4(–) CD8a(–) CD10(–) CD14(–) CD20(–) CD24(–) CD31(–) CD34(–) CD38(–) CD41a(–) CD45(–) CD49b(–) CD41/61(–) CD62p(–) CD73(–) CD90(–) CD133(–) MHC–II (–)	CH-OST	(173)
DMEM-HG + 10%FBS + 1% antibiotic-antimycotic + 1% Glutamax + 10ng/ml bFGF	UC	2x10^4^ cells/cm^2^	CD105^a^(+) CD105^b^(–) CD105^c^(–) CD73^a^(+) CD73^b^(–) CD73^c^(–) CD34(+) CD44(+) CD90(+) CD14(–) CD45(–)	AD-CH-OST	(174)
aDMEM + 10%FBS + 1% pen/strep	AT	NK	CD90(+) CD44(+) CD29(+) CD45(–)	AD-CH-OST	(175)

*DMEM, Dulbecco's Modified Eagle Medium; DMEM-LG, Dulbecco's Modified Eagle Medium—Low Glucose; DMEM-HG, Dulbecco's Modified Eagle Medium—High Glucose; aDMEM, advanced Dulbecco's Modified Eagle Medium; FBS, fetal bovine serum; Pen, Penicillin; Strep, Streptomycin; L-glut, L-glutamine; bFGF, basic fibroblast growth factor; CD, cluster of differentiation; AT, adipose tissue; BM, bone marrow; UCB, umbilical cord blood; WJ, Wharton's jelly; UC, umbilical cord; AD, adipogenic differentiation; CH, chondrogenic differentiation; OST, osteogenic differentiation; CD105^a^, CD105 antibody clone OTI8A1; CD105^b^, CD105 antibody clone SN6; CD105^c^, CD105 antibody clone P3D1; CD73^a^, CD73 antibody clone 7G2; CD73^b^, CD73 antibody clone AD2; CD73^c^, CD73 antibody clone D12; MHC-I, major histocompatibility complex-I; MHC-II, major histocombatibility complex-II; (+), positive expression; (+/–), mild expression; (–/+), moderate expression; (–), negative expression; /, not assessed; NK, not known*.

**Table 4:** Characterization of canine mesenchymal stromal cells cultured in serum-free media.

**Culture media**	**Tissue source**	**Seeding density**	**Surface markers**	**Tri-lineage differentiation potential**	**References**
Commercial SFM-StemPro®	AT	5 × 10^3^ cells /cm^2^	CD90(+) CD44(+) CD54(+) CD45(–) CD34(–) MHC–II(–)	/	(151)
In-house SFM	AT	3 × 10^5^ cells /cm^2^	CD105(+) CD90(+) CD14(–) CD34(–)	AD-CH-OST	(176)
In-house SFM and Commercial SFM—RoosterBio	AT	3 × 10^5^ cells /cm^2^	CD44(+) CD90(+)	AD-CH-OST	(150)

*SFM, serum-free media; AT, adipose tissue; CD, cluster of differentiation; AD, adipogenic differentiation; CH, chondrogenic differentiation; OST, osteogenic differentiation; (+), positive expression; (–), negative expression; /, not assessed; NK, not known*.

The most common basal media used are Dulbecco's Modified Eagle Medium (DMEM) in low (LG) and high (HG) glucose formulations, followed by Minimum Essential Medium α (α-MEM) which contains a higher content of non-essential amino acids, sodium pyruvate and vitamins compared with DMEM. Reports have shown that α-MEM is superior compared to DMEM for *in vitro* expansion of bone marrow (BM)—murine and equine derived MSCs, although with contrasting results on their differentiation ability (145, 146). This was also the case for canine MSCs that required the supplementation of basic fibroblast growth factor (bFGF) to achieve a similar effect (147). Phenol red-containing media is commonly used in human and animal MSCs manufacturing mostly regarded as a pH indicator to monitor the cell culture conditions. It seems to have as well an effect on different types of cell differentiation, as it inhibits chondrogenic differentiation and affects osteogenic differentiation (148). Based on this it should be used with caution when cells are differentiated *in vitro*, however it does not seem to affect to a larger extent routine cell proliferation and growth being a weak estrogen (149).

FBS is the main media supplement used in concentrations from 10 to 20%, with varying concentrations of additional supplements such as bFGF (1–10 ng/ml), glutamine (2–2.5 mM or 1% L-glutamine), antibiotics in form of penicillin (100 U/ml), streptomycin (100ug/ml) or combined as 1% penicillin/streptomycin, as well as gentamicin (80 ug/ml) and in some cases antifungal agents such as Amphotericin B (Fungizone) at a concentration of 1.25 ug/ml or 0.05%. This massive variability in the starting cell culture conditions was additionally increased by different cell seeding densities starting from 100 up to 30,000 cells/cm^2^ which strongly influences the growth potential of the cells. In our experience, high cell seeding density is needed in the starting culture passages due to the fact that the cells have been recently isolated from a complex three-dimensional environment and close cell-to-cell contact is needed in order for them to initiate *in vitro* growth efficiently in a very different planar two-dimensional system. Different cell seeding densities therefore impact the overall growth dynamics of MSCs and should be recognized as important parameters that need to be standardized within the manufacturing process. Additionally, currently in the literature it is common for researchers to state the cell passage number to identify the times the cells have been subcultured *in vitro*. This indicates the timeline of *in vitro* processing and is used as a standardized comparison of cells used at similar passages across different *in vitro, in vivo* or clinical applications. However, it could be the case that different cell seeding densities, as well as intrinsic and extrinsic factors could influence cell proliferation therefore it is questionable to what extent the cell passage could useful for this purpose. From this perspective, to be able to standardize the process it would be more appropriate for the authors to report the population doubling level instead of the cell passage number.

Progress has been made in veterinary medicine regarding formulation and testing of serum-free media alternatives. Recently, a group from the Center of Veterinary Medicine of the US Food and Drug Administration (FDA) have reported the efficacy of a newly developed-serum free medium for canine MSCs, successfully optimized with a cocktail of growth factors that achieved comparable results to FBS supplemented media (150). Interestingly, they also tested a commercially available SFM for human MSCs that failed to support cell growth, which is in contrast to other studies reporting successful cell growth and proliferation and alterations in immune modulation abilities of canine and equine MSCs cultured with human serum-free media (151). This indicates that tailored species-specific media are needed to have optimal condition for MSC expansion. The process of designing and developing a species-specific media can be challenging due to the high costs and potential lack of species-specific growth factors. For this aim, potential collaborations with growth media manufacturers could provide a valuable cost-effective alternative and assistance in the process of cell media development to address challenges in animal cell manufacturing.

### Cell Expansion and Banking

Once the stromal vascular fraction or bone marrow concentrate have been obtained, they are subsequently seeded in tissue culture flasks for *in vitro* expansion. This is essential as the number of endogenous MSCs present in these populations are very low, and to meet clinical needs these need to be further expanded *in vitro*. This is usually performed until a significant number of cells is obtained, usually until passage 4–5 (P4–P5) after which canine MSCs are known to undergo senescence (119). The culture expansion process allows time to obtain a potentially homogeneous population in terms of size, shape and morphology characterized by its proliferation ability, differentiation capacity and immune phenotype identity (152). Different expansion strategies can be used and optimized to avoid the risk of overexpansion in relation to the needed batches. For therapeutic use on a case-to-case basis of lower cell numbers, cells can be cultured in standard two-dimensional planar flasks or small sized bioreactors. The conditions can be further adapted with the addition of growth factors such as FGF2, PDGF, EGF, known to maintain the self-renewal state of MSCs, increase their proliferation and delay senescence (153), or in alternative it has been reported that expansion time can be reduced when MSCs are cultured in hypoxic conditions with supplementation of PL (154). For high cell numbers needed for large clinical trials, it would be optimal to use advanced manufacturing systems such as stirring bioreactors with the potential use of microcarriers that would allow a standarised expansion of the cells (155).

In this context, the need for specialized equipment, providing a safe and sterile environment, and trained stem cell technologists necessitates a collaboration between research laboratories with veterinary surgeons for the supply of animal tissues and production of MSCs. Given the fact that in human medicine the median clinical dose of injected MSCs is 10 × 10^6^ cells/patient (156), cell production for clinical use requires an extensive *in vitro* expansion process comprised of cryopreservation steps in liquid nitrogen respecting good laboratory practices of slow freezing and fast thawing. This multistep procedure involves generating a master cell bank (MCB) consisting of primary isolated, expanded and cryopreserved cells at early cell passage (P1) representing the main cell reservoir. As mentioned previously, MSCs need to be expanded extensively to obtain high cell numbers and for this purpose samples from the MCB are thawed, cultured *in vitro* over several passages, characterized and cryopreserved as an intermediate or working cell bank (WCB). In moment of need the WCB will be thawed as the final product for clinical use. This strategy is used in both autologous and allogeneic clinical settings. In the former, it allows the generation of a cell bank which can be used over a lifetime for expansion and multiple treatments, similar to human umbilical cord blood banks that have existed for more than 20 years (157, 158). Similarly, in the allogeneic setting it allows the choice of an approriate young and healthy donor as an ‘off-the-shelf’ product that has been highly characterized and readily available for therapeutic use, allowing sufficient time to organize the treatment between researchers, surgeons and pet owners (159).

To be able to meet the demands of allogeneic cell products, significant efforts are being made to upscale the production of human and equine MSCs (160, 161) by increasing the surface area using multilayered flasks, microcarriers and bioreactors. In the context of canine MSCs, cells seeded in HYPERflask™s retained a similar phenotype, proliferation ability and immunomodulatory action, whilst producing a higher number of viable cells compared to standard tissue culture flasks (151). This demonstrates a proof of concept and future studies are needed to explore more advanced manufacturing systems that will allow standardization and acceleration of the manufacturing process of canine MSCs.

### Release Tests

#### Sterility Test

Considering the significant commercial interest in producing MSCs for veterinary clinical use, it is paramount that manufacturers adopt a series of protocols that will identify the critical quality control points. For each of those quality standards, a risk assessment analysis should be performed with an appropriate contingency plan to prevent any disruption in the production process. The complexity in the manufacturing of living cells that cannot be terminally sterilized and filtered, highlights the need for *in process* and final product control of microbiological contamination (endotoxins, adventitious viruses, bacteria and mycoplasma) with sterility being fundamental for the quality and efficacy of MSCs. *In process* controls of sterility are based on macroscopic, microscopic and molecular evaluations at critical points of the manufacturing process. These include the tissue/tissue fluid prior to processing, all media and corresponding supplements before use in cell culture, as well as prior to the cryopreservation and packaging of the final product. At a macroscopic level, the color of the media and level of opaqueness are good indicators of possible sterility issues, which can be confirmed with microscopic evaluation. Additionally, the morphology of the cells needs to be carefully monitored as MSCs display a typical fibroblast-like shape morphology. The presence of detached or altered cells in terms of size and shape, might be an indication of cell senescence, apoptosis or contamination, and in that case the cells should be monitored and if problems persist accordingly discarded.

Antimicrobials are used in the early stages of manufacturing, but their use should be avoided in the later stages, as residues could otherwise be left in the final product (157, 162). The shelf-life duration of allogeneic products needs to be determined through batch-to-batch stability studies and in general is limited (159). Given the short shelf-life of MSCs between cell thawing and clinical application, sterility methods defined by the European Pharmacopoiea are inadequate in terms of timing intervals because they require a detection time of up to 48 and 72 hours, and any results obtained prior are not fully conclusive. For this reason, the FDA has evaluated several rapid tests for the detection of microorganisms such as BacT/ALERT™ (Biomerieux), Rapid Milliflex™ (Merck Millipore) and BACTEC™ (Becton Dickinson), but the results can take up to 72 h, which is still outside of the optimal time range. To overcome this, a combined approach between Gram staining and rapid microbiological tests is currently used in Europe, but there is the need to further refine this screening. It is therefore fundamental to respect the critical in process controls and validate the manufacturing procedures, considering that the screening results of the final product will most likely be obtained after the cells have been injected (163). In this scenario the advantage of using allogeneic cell products, previously screened and tested from a controlled and standardized master cell bank is an advantage, compared to autologous cell products manufactured on demand where the speed of the process is essential for the treatment of the patient.

#### Cell Characterization

For the purpose quality control, it is vital to define critical attributes of the final cell product. These attributes include indicators of cell phenotype, identity and purity. Historically, the minimal criteria that defined human MSCs *in vitro* included: plastic adherence, fibroblast-like morphology, capacity to undergo tri-lineage differentiation (adipogenic, osteogenic and chondrogenic) and the expression of a set of positive (CD105, CD73, CD90) and negative (CD45, CD34, CD14 or CD11b, CD79α or CD19, MHC-II) surface markers (114, 164, 165). In general terms, animal-derived MSCs have been characterized using the same criteria as human MSCs. Here we will focus mainly on cell surface marker expression as a characterization assay to be used routinely in cell manufacturing.

The majority of studies involving veterinary applications have used a combination of antibodies with different species-specificity (Tables 2–4), where the most common set of positive cell surface markers investigated are: CD90, CD44, CD29, and CD73, whilst CD45 and CD34 are routinely used as negative MSCs markers. There are few studies that selected CD4 or CD33 as a marker to identify canine MSCs, but these have shown variable experimental results (20, 173, 177). However, simply replicating a human panel of criteria to define cells from other species is not sufficient, as some markers might not be adequate and there may be others more appropriate markers that have not yet been identified.

When SVF was isolated from periovarian tissue, it was found to have a higher percentage of CD90, CD44 and CD29 positive cells, compared with subcutaneous fat and falciform ligament derived SVF which could correlate to the degree of vascularisation (99). However, expanded adipose tissue-derived MSCs (AT-MSCs) in consecutive passages up to passage 8 (P8), isolated from subcutaneous and periovarian tissue showed no differences in expression of CD44, CD45 and CD90, which could be the result of *in vitro* expansion in standardized conditions (102). For example, the extent of vascularisation of adipose tissue and harvest technique has been reported to influence the expression of CD34 (90) given the fact that these cells are located in the periendothelial niche (178). Additionally, it has been shown that markers like CD73, which is one of the defining positive markers for human MSCs, has a low or absent expression in canine MSCs. Divergent results on the CD73 marker is found by Kang et al. (CD73 positive) and Seo et al. (CD73 negative) studies, both of which cultured umbilical cord blood derived MSCs in Dulbecco's Modified Eagle Medium (DMEM) contained medium (170, 173). Others have demonstrated a variation in CD73 expression in early and late culture passages of canine BM-MSCs, with a significant decrease of expression in late phases. This indicates that extensive *in vitro* expansion is a factor conditioning the surface marker profile of MSCs (179), which could be intuitive given the fact that cells are isolated from a complex 3D environment rich with intercellular and paracrine stimuli, to a planar 2D environment resulting in a homogeneous cell population in a controlled environment.

A major challenge relates to the lack of validated species-specific reagents. Such reagents are commonly available for human and laboratory animal-derived MSCs but are unavailable or unvalidated for other veterinary species (180). The cluster of differentiation (CD) nomenclature was first introduced in 1993 for canine antigens, when human and murine homologs were identified on canine leukocytes, indicating that cross-reactive antibodies could potentially be used for canine cell characterization (181). Similarly, the same antigen-antibody approach was translated for other surface markers including those identifying MSCs, where currently the use of human and murine cross-reactive antibodies is applied in absence of canine species-specific antibodies. The manufacturing of species-specific antibodies is a cumbersome and expensive process, therefore the use of canine cross-reactive antibodies would be the most likely choice used by researchers in the absence of readily available canine specific antibodies (181). To identify the homology of human and canine CD markers, in terms of antibody binding abilities, a Basic Local Alignment Search Tool (BLAST) analysis comparing the simple text format for storing aminoacidic sequence (FASTA) of human and canine extracellular domains of the CD markers used for MSCs characterization can be performed (182–185). Therefore to be able to confirm the validity of the surface marker expression results when using a non-species-specific antibody, a BLAST analysis of the immunogen sequence of the antibody with the target epitope could provide insights into possible antibody reactivity.

It is good practice to test antibodies for canine cross-reactivity prior to their application in MSC characterization. Rozemuller and colleagues tested a panel of human monoclonal antibodies designed to identify a set of clonogenic MSCs in primary cells from human bone marrow (186), on *in vitro* expanded canine MSCs. This panel included antibodies to the surface markers W5C4, CD276, CD56, W4A5, MSCA-1, CD146, CD349 and CD271. On the contrary, Tryfonidou et al. have demonstrated that the commercial anti-human CD276 antibody (clone FM276) was not able to bind to canine CD276 (187), suggesting that it is essential to select specific validated antibody clones that have cross-reactivity with canine cells.

Efforts have been made to characterize canine MSCs using species-specific antibodies for CD29, CD44, CD73, CD90, CD34, CD45 and MHC-II, with the aim of performing cell characterization studies that will not be hindered by potential cross-reactivity issues (173, 188). This approach can be useful for routine characterization of canine MSCs, however it has technical limitations due to a restricted panel of commercially available canine specific antibodies.

In canine OA clinical studies, the characterization of MSCs immunophenotype is most often regarded as a routine protocol based on a minimal standardized conventional set of set of positive (CD44, CD29, CD90) and negative surface markers (CD34, CD45, MHC-II) (8, 9, 29, 31, 34). From this practical perspective, immunophenotyping is used to demonstrate the surface identity of the cells manufactured in different facilities worldwide, which however does not correlate with the functionality and therapeutic efficacy of the final product. A major challenge in human and veterinary medicine remains the fact that these criteria define MSCs based on their *in vitro* properties, and while it contributes to a defined characterization among different research groups, it is not fully known what is the functional role of these markers and whether new markers are needed that would be able to predict the *in vivo* function of MSCs.

#### Potency Assay

Based on the European Guidelines on Human Cell-Based Medicinal Products, a potency assay is a “quantitative measure of biological activity based on the attribute of the products that ideally should be linked to the clinical response” and can be performed *in vitro* using cell assays or *in vivo* on pre-clinical animal models (189, 190). One of the major challenges that impacts successful clinical translation of MSC therapies relates to the fact that the *in vivo* pharmacological and biological activity of transplanted MSCs is still poorly understood. This remains a significant hurdle for researchers, regulatory agencies and commercial organizations. Ideally, potency assays for MSC-based products would be quantitative laboratory tests that would predict the therapeutic activity of the cell product, thereby acting as a product release test for quality assurance. It is expected that potency assays would be disease-specific. The complex biological nature of a living cell product, uncertainties surrounding the nature of engraftment and persistence in the transplanted environment as well the lack of understanding of the therapeutic effects present great challenges in defining an effective potency assay for MSCs (190, 191). At this time, it is understood that MSCs exert their therapeutic effects by release of secreted factors to the host. The identification of secreted factors and the assessment of their effect on the injured microenvironment may allow the development of meaningful potency assays (158, 192).

For the treatment of canine OA, the therapeutic activity of MCSs is mainly mediated by immunomodulatory interactions with the innate and adaptive immune system as well as through trophic and paracrine effects that ultimately counteract synovial inflammation and secrete chondroprotective factors promoting cartilage repair (193).

Based on the proposed mechanisms of actions, several potency assays can be considered. The first one involves a quantitative determination of prostaglandin E2 (PGE2) concentration in cell culture supernatant. According to several published studies, PGE2 derived from MSCs promotes the transformation of pro-inflammatory M1 to anti-inflammatory M2 macrophage polarization (194) and inhibits natural killer (NK) cell-mediated cytotoxicity (195). In addition, PGE2 produced by MSCs suppresses the production of IFNγ in T helper type 1 (Th1) cells and promotes the proliferation of Tregs, inhibiting T cell proliferation and cytokine production (196). Chow et al. have shown that T cell suppression was mediated PGE2 produced by canine MSCs. In this *ex vivo* study, canine AD-MSCs and BM-MSCs were cocultured with canine T cells and the immunomodulatory action was found to be mediated *via* TGF-ß pathway activation and secretion of PGE2 resulting in inhibition of T cell proliferation (197). In recent times the PGE2 assay has been used as a clinically relevant potency test for an approved equine MSC product used for joint disease treatment (131). Another option is a quantitative determination of indoleamine 2,3-dioxygenase (IDO) secretion in a mixed lymphocytes reaction (MLR) assay. MSCs secrete IDO inducing M1-to-M2 polarization and T-cell immunosuppression as well through catalyzing tryptophan degradation into immunoregulatory kynurenine and Vav1 signaling (198, 199). Kang et al. have demonstrated that IDO derived from canine AD-MSCs suppresses leukocyte proliferation effectively (200).

Besides the immunomodulatory approach other potency assays can be considered. To improve the clinical outcome, MSCs can be differentiated *in vitro* into chondrogenic lineage, that can be assessed by the quantitative determination of the chondrogenic marker cartilage oligomeric protein (COMP), aggrecan and collagen type 2 on MSC products by RT-qPCR (201). To study the cross-talk beween MSCs and OA chondrocytes and synovial cells respectively, transwell co-cultures represent an efficient *in vitro* system able to mimic to some extent the joint environment. Canine lentiviral transduced MSCs expressing PDGF and heme oxygenase-1 (HO-1), were co-cultured with lipopolysaccharide (LPS)-stimulated canine chondrocytes and synovial cells using this system. Pro-inflammatory and pain-related molecules (matrix metalloproteinase 13; nerve growth factor) expression were downregulated, and genes involved in synthesis and maintenance of the extracellular matrix (aggrecan, collagen type 2) were upregulated in the co-cultured chondrocytes, ultimately contributing to re-establishment of the joint homeostasis (202).

Although the research data of canine chondrogenic induced MSCs (ciMSCs) in the treatment of joint diseases are limited, a large number of equine-related studies have proven that ciMSCs promote cartilage repair through producing glycosaminoglycans, aggrecan, and collagen type 2 (203–205) and gene expression of a chondrogenic marker of cartilage turnover was used to determine the potency of the EMA authorized equine MSCs product (130).

The ultimate challenge that remains to be addressed is to demonstrate a functional relationship between the potency assay result and the observed clinical outcome, ultimately ensuring consistent product quality (159). This highlights the importance of performing properly designed clinical trials to validate these tests.

### Transport and Cell Delivery

MSCs are highly sensitive to environmental cues and optimal conditions are required to maintain their stability, viability and sterility. Based on this, the final product formulation and transport to the veterinary clinic need to be highly controlled to maintain the quality chain between the cell manufacturing process and delivery to the patient under good clinical practice (GCP) procedures. MSCs can be transported from the laboratory to the delivery site at room temperature, chilled at 2-8°C or frozen, which will vary in relation to the proximity between the facilities. Many published canine clinical studies have been developed in academic settings where cells are readily thawed, washed and prepared for the surgeon to be injected at room temperature (Table 1). In the context of clinical cell manufacturing, usually cells need to be transported long distance and up to 24hrs they can be stored at 4–8°C, however for long-term the cells need to be shipped frozen for optimal viability (206). The latter condition will maintain the best the stability of the product, however most likely it will be the duty of the veterinary surgeon to prepare the final formulation which adds complexity and potential alteration of the cell product as it can be potentially mishandled.

Based on the published literature for canine clinical trials the delivery vehicles used for injection of MSCs for canine OA could be classified broadly as chemical and biological solutions. These include respectively PBS and sterile saline solution for the former, and PRP and hyaluronic acid and salt for the latter. Cells suspended in these carriers were delivered in different volumes ranging from 0.3 ml up to 1–2 ml for intra-articular and 10–20 ml for intra-venous injection (Tables 5, 6). The advantages in using PBS and saline solution are mainly due to their standardized composition, broad availability in the manufacturing and clinical setting and the flexibility to be used both for systemic and local MSCs delivery. The use of biological carriers such as PRP and hyaluronic derivates, especially for treatment of osteoarthritis can be beneficial as they can contribute to the joint healing process. In OA patients, there is a reduction of HA concentration in the joint, altering the composition of the synovial fluid and affecting the joint mobility (207). Similarly, the concentrated platelet-derived factors as constituents of PRP can promote cartilage healing and enhance anti-inflammatory responses thereby amplifying the therapeutic effects of MSCs (208). The storage however of MSCs in biological carriers has to be further evaluated as it has been demonstrated that a gradual time-related degradation of their components such as growth factors and cytokines can affect and alter ultimately the viability of MSCs (206). In addition, 5% dextrose solution (5%DS), heparin in saline (Hepa-Sal) and Hartmann's solution (HS) have also been tested *in vitro* as potential carriers for canine MSCs, showing differences in terms of cell viability and proliferation. Saline solution and 5%DS were able to maintain optimal viability and colony forming units ability up to 12hrs. Gene expression levels of CD105 and CD90 reached significantly higher expression in cells stored in PBS, while collagen type II alpha 1 chain (COL2A) and SRY-Box Transcription Factor 9 (Sox 9) were highly expressed in chondrogenically-differentiated MSCs that had been stored respectively in saline and HS (209). Based on this, it is important to carefully consider and test various cell carriers by testing their efficacy and stability on the final product of the manufacturing process.

**Table 5:** Chemical vehicles used for delivery of MSCs in canine OA.

**Vehicle**	**Volume**	**Delivery**	**References**
PBS	/	IA	(39)
D-PBS	0.3 ml	IA	(9)
D-PBS	0.5 ml	IA	(31)
PBS	/	IA	(78)
PBS	0.6 ml	IA	(7, 25)
PBS	1 ml	IA	(35)
PBS	1 ml	IA	(74)
PBS	1 ml	IA	(70)
PBS	0.5 ml	IA	(84)
D-PBS + HS 200 IU	10–20 ml	IV	(33)
Sterile saline	0.5 ml	IA	(73)
0.9% saline	/	IA+IV	(37)
0.9% saline	0.5 ml	IA	(79)
0.9% saline	0.6 ml	IA	(34)
0.9% saline	1 ml	IA	(69)

*PBS, phosphate-buffered saline; D-PBS, Dulbecco's phosphate-buffered saline; HS, heparan sulfate; IU, international unit; IA, intra-articular; IV, intra-venous*.

**Table 6:** Biological vehicles used for delivery of MSCs in canine OA.

**Vehicle**	**Volume**	**Delivery**	**References**
0.5% sodium hyaluronate	/	IA	(76)
10 mg/ml HA + PBS	0.6 ml	IA	(66)
20 mg/2 ml HA	/	IA	(27)
HA	0.5 ml	IA	(84)
0.5% sodium hyaluronate	1 ml	IA	(83)
PRP	1–2 ml	IA	(36)
PRGF (Endoret)	4 ml	IA	(67)
PRP	0.5 ml	IA	(75)
PRP	1.5 ml	IA	(86)

*HA, hyaluronic acid; PBS, phosphate-buffered saline; PRP, platelet rich plasma; PRGF, plasma rich in growth factors; IA, intra-articular*.

## Conclusions and Future Directions

Over recent years, the interest in using stem cells therapies for treatment of diseases in veterinary patients has been noteworthy, stimulating research and development of cell-based products worldwide. The veterinary cell therapies field is progressing. Firstly, EMA has already approved the marketing authorization for two MSC-based equine products, showing the need of introducing cell-based products for routine veterinary medicine clinical applications. Based on the current classification of animal species by the European Medicines Agency (EMA), dogs are classified as major species, while horses belong to the minor species category. The different categories imply also different routes of approval and marketing authorization obtainment, as equine products can undertake the minor use/minor species (MUMS) route (210). Indeed there is almost a two-fold higher number of registered medicinal products for dogs, compared to ones for the horses based on the latest EMA records (211). Secondly, dogs as pets are frequently considered “family members” and their owners often seek the best treatment with advanced products, therefore expanding consumer need. Finally, dogs represent a good translational animal model due to their similarities with humans, the human genome and disease conditions. Therefore manufacturing of standardized canine cell products will foster a “one medicine” approach to the study of differences and similarities between the two species. In general, there is need of additional scientific and clinical knowledge for canine MSCs to be able to reach the market, as the regulatory frameworks and guidelines are being shaped at the moment. Incentives in terms of grants for large cohort clinical studies that would give time-related information are critical to generate safety and efficacy data. These need to be further standardized since multiple injections might be needed to compensate for the possible low survival rate of the injected cells and the timing between treatments needs to be optimized as well. So far, the margin of safety for canine MSCs product seems to be very high with no adverse effects reported in canine patients in clinical studies. The development of valid functional potency assays that can be translated into a desired clinical outcome, will be of great benefit for the integration of MSCs in the current therapeutic protocols and their introduction in the clinical practice. This will be also helpful in defining release criteria standards that can be beneficial for cell manufacturers and for the regulatory bodies during the approval process. It is important for the identification of anomalous batches and would pave the way toward a successful translation in the veterinary clinics with defined SOPs and quality controls. Ultimately this will lead to clinical trials representing a central element for this progress that provide valuable input on dosage, safety, efficacy and setup of an efficient pharmacovigilance system.

Compared with human medicine the number of animal cell products is very limited and highlights the complex pathways, considerable financial investment, expertise and time to manufacture GMP-grade MSCs that are a current limiting factor in veterinary medicine. However, animal cell therapies should be manufactured following GMP standards and several considerations can be made on how this can be facilitated in terms of costs. In the context of the One Health initiative, one approach would be for alignment of resources in both human and animal cell product manufacturing. In this way, general maintenance costs, as well as control of raw materials, outsourcing services, and know-how can be shared between facilities allowing better control of costs and financial viability. From an economic perspective, in human medicine the burden on the healthcare system represents an important baseline for budget distribution and future drug development. This can also be considered on the veterinary side and potentially offer major financial support for the development and manufacturing of cell therapies. Another possible approach would be to consider building veterinary hospital GMP facilities, since they will significantly reduce associated costs with shipment of raw materials and delivery of cell products, as well as offer a personalized approach in direct communication with the veterinary surgeons. The manufacturing process can be developed in timely manners for optimal application in clinical trials or single clinical patients. Moreover, it will be a possibility for veterinary surgeons to get basic training in cell technologies and have a major awareness on the correct use of cell therapies.

A major challenge in cell manufacturing is the intrinsic heterogeneity that MSCs display on multiple levels (donor-, tissue-, cell-related) across all species and these different phenotypes may translate in potentially different functions. However, based on the current knowledge, several considerations can be made.

First of all, the routine analysis of the expression of CD markers has been recommended (114, 212) and should be performed to be able to define the multipotent identity and generate an immunophenotypic profile. For canine MSCs as mentioned extensively there is a need to identify optimal species-specific cell surface markers. However, concentrating a population of MSCs based on the cell surface antigens might result in a functionally heterogeneous population (213, 214) which is not desired in the context of clinical cell manufacturing.

In this setting, it is important to demonstrate homogeneity in the biological read-out used as release criteria for the treatment of the targeted disease. The intrinsic heterogeneity of MSCs plays an important role for their *in vivo* function, as it was suggested that different cell populations may get activated by various stimuli and contribute accordingly to resolution of the insult (215). Cell manufacturers could take advantage of this ability and pre-treat MSCs with different stress-induced stimuli to try to reduce or abolish any heterogeneity in their functions. For example, it has been reported that pre-treating MSCs with pro- inflammatory molecules reduces heterogeneity in their immunosuppressive abilities (216).

From a technical perspective, culturing the cells in a traditional planar 2D systems might result in induced heterogeneity as each flask represents a different environment, can be potentially subject to batch-to-batch and operator-related variability (158). In alternative, scale-up systems could be a feasible alternative for standardization of the process, while obtaining a higher number of cells with a stable phenotype in controlled environment (217). Based on this further studies are needed to explore the effects of small-sized and large-scale manufacturing of canine MSCs as well as the induction of any cell-adaptation mechanisms that might induce cell heterogeneity.

In the clinical setting, the use of auto-MSCs versus allo-MSCs would be decided based on practical considerations on a case-to-case choice, as well as the current available clinical data to confirm the optimal therapeutic approach. Allogeneic products undergo all the phases of the cell manufacturing process starting from careful donor selection, microbiological controls, in process controls of cell morphology, cell surface marker expression, potency assays and sterility testing that would result in high-quality, well-standardized cell products with a higher margin of safety. In advanced-age canine patients and/or affected by comorbidities the use of off-the-shelf allogeneic products from young and healthy donors would be beneficial as these cells show higher proliferation and growth rates. Where multiple injections are needed, readily available allogeneic products would be more convenient compared to autologous MSCs. From a clinical perspective, autologous MSCs could imply multiple sampling procedures to obtain sufficient number of cells for the intended clinical application. However, it is important to note that there is need of long-term large clinical studies to demonstrate the safety and efficacy of allogeneic MSCs, especially for multiple injection clinical studies where the formation of alloantibodies should be assessed.

Both studies with positive and negative outcomes need to be published and shared, as this would allow the opportunity to define better the therapeutic efficacy of canine MSCs.

To be able to progress with canine MSC products, a list of regulatory and scientific guidelines is needed that would define the manufacturing and clinical process in a constructive manner with inputs from cell biologists, cell manufacturers, veterinary surgeons and regulatory agencies. We suggest the following recommendations to be considered by the relevant stakeholders:

- Recommendations for cell manufacturers:
  1. Future detailed studies are needed on the identification and characterization of canine specific cell surface markers.
  2. Consistent validation of reagents used, especially if they are not species-specific by using appropriate positive and negative control.
  3. Efforts are needed for the design of canine-specific, chemically-defined culture media that will contribute to standardization of manufacture.
  4. The manufacturing process is crucial for the product. Inevitably there will be operator-to-operator variability, however, a standardization of quality control parameters, including a desired value of a potency assay could be a common target to be pursued for a specific disease by each laboratory to obtain a valid therapeutic product.
  5. Publish and report results at all relevant stages (*in vitro, in vivo* and at the clinical stage), which is critical to get critical data for the advancement of the field.
- Recommendations for veterinary surgeons:
  1. Ask for a quality control assurance certificate from your cell provider including cell numbers, viability, and microbiological control.
  2. Inform yourself about the scientific publications specifically related to the product of interest.
  3. Create a standard operating procedure (SOP) within your clinical practice establishing a routine reproducible procedure for the sample collection in case of autologous cell products, allowing repeatability and minimize inter- and intra-operator variability.
  4. Select a preferential site of sample collection that would consider autologous or allogeneic use, as well as include breed characteristics, gender and operation procedure to facilitate the process and reach consistency by respecting good clinical practices in avoiding contamination.
  5. Data on safety and efficacy is currently needed for wider applications of cell therapies – to this aim perform objective clinical assessment such as goniometric measurements of the range of motion and force plate analysis (212), and collect data from owners by using the Liverpool Osteoarthritis in Dogs (LOAD) or Canine Brief Pain Inventory (CBPI) to create a standardized and valid pharmacovigilance system.

Cell therapies are considered disruptive technologies and this is particularly evident as they can still be considered experimental to a certain level in an environment where more evidence on the subject is needed. Education and training are very important and only educated professionals should use cell therapies as they would also possess the background knowledge on understanding important up-stream parameters outlined in the present review. In order to successfully do the next step, collaboration efforts are needed at the moment between general practitioners, specialists and stem cell scientists as a forum where knowledge, know-how and current challenges can be addressed. In particular they could represent a professional body that can communicate with regulatory agencies and healthcare authorities, highlighting the real challenges and offering solutions that can be adapted for biological products. In human medicine global initiatives have been established in Europe (218) and Australia (219) as platforms for dissemination of pre-clinical, clinical and regulatory information among the main stakeholders involved. Similar initiatives should be considered in the field of veterinary medicine that would also include animal owners as an important factor in the follow-up and outcome assessment of therapy.

In the near future, the number of canine MSC-based products on the market will most likely increase, as this technology will continue to grow and develop. It is therefore vital that manufacturing procedures are highly controlled during the research and development of such products, encouraging reproducible high standards of manufacture so that clinical outcomes can be reliably evaluated.

## Author Contributions

AI and FB contributed to the writing and conceptualization of the manuscript. MW contributed to the literature revision and manuscript writing. TA designed the figures and with GS contributed to critical revision from a laboratory and manufacturing perspective. JA, SB, AB, RC, LC, JD, SG, RH, AM, and RM critically reviewed the manuscript. All authors contributed to the article and approved the submitted version.

## Funding

The Celtic Advanced Life Science Innovation Network is an Ireland Wales 2014–2020 Programme Part Funded by the European Regional Development Fund Through the Welsh Government.

## Conflict of Interest

JA and RH were employed by ValleyVets. SB was employed by Weighbridge Referral Centre. AB was employed by BrayVet. RC was employed by Alphavet Veterinary Centre. LC was employed by Veterinary Specialists Ireland. JD was employed by ArkVets Galway. SG was employed by Gilabbey Veterinary Hospital. AM was employed by Northern Ireland Veterinary Specialists. The companies were not involved in the study design, collection, analysis, interpretation of data, the writing of this article or the decision to submit it for publication. The remaining authors declare that the research was conducted in the absence of any commercial or financial relationships that could be construed as a potential conflict of interest.

## Publisher's Note

All claims expressed in this article are solely those of the authors and do not necessarily represent those of their affiliated organizations, or those of the publisher, the editors and the reviewers. Any product that may be evaluated in this article, or claim that may be made by its manufacturer, is not guaranteed or endorsed by the publisher.
